# Combined treatment with endobronchial Watanabe spigot and *N* ‐butyl‐2‐cyanoacrylate for refractory pneumothorax in COVID‐19

**DOI:** 10.1002/rcr2.923

**Published:** 2022-03-13

**Authors:** Chie Morita, Atsushi Kitamura, Kohei Okafuji, Shosei Ro, Ryosuke Imai, Kasumi Shirasaki, Yu Watanabe, Naoki Nishimura

**Affiliations:** ^1^ Department of Pulmonary Medicine Thoracic Center, St. Luke's International Hospital Tokyo Japan; ^2^ Emergency and Critical Care Medicine St. Luke's International Hospital Tokyo Japan

**Keywords:** COVID‐19, endobronchial Watanabe spigot (EWS), *N*‐butyl‐2‐cyanoacrylate (NBCA), pneumothorax

## Abstract

Coronavirus disease 2019 (COVID‐19) causes pneumothorax or mediastinal emphysema in approximately 1% of patients. According to the British Thoracic Society guidelines, the next treatment option for patients with persistent pneumothorax despite chest drainage is pleurodesis or surgery. In fact, there are reports of autologous blood pleurodesis or surgery for the treatment of pneumothorax caused by COVID‐19. However, elderly patients or patients in poor general condition may not be able to tolerate surgical invasion. In this report, we present two patients who did not respond to chest drainage or pleurodesis and who were not suitable for surgery because of their poor general condition. These patients were successfully treated with an endobronchial Watanabe spigot and *N*‐butyl‐2‐cyanoacrylate. This method may be an option for the treatment of refractory pneumothorax in COVID‐19.

## Graphical abstract

In this report, we describe, for the first time, two cases of pneumothorax refractory to chest drainage both before and after positive pressure ventilation in patients with coronavirus disease 2019 who were successfully treated with an endobronchial Watanabe spigot and *N*‐butyl‐2‐cyanoacrylate.

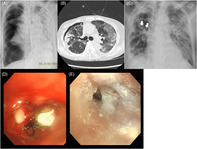

## INTRODUCTION

The incidence of pneumothorax and mediastinal emphysema in patients with coronavirus disease 2019 (COVID‐19) is approximately 1%,^1^, ^2^ and the probability of developing pressure injuries, such as pneumothorax and mediastinal emphysema, in ventilated patients has been reported to be 15%.^3^ Previous reports indicate that pneumothorax develops in patients with COVID‐19 who do not require positive pressure ventilation, suggesting that multiple mechanisms, in addition to pressure trauma, are involved in the development of pneumothorax in COVID‐19.^1^

In this report, we describe, for the first time, two cases of pneumothorax refractory to chest drainage both before and after positive pressure ventilation in patients with COVID‐19 who were successfully treated with an endobronchial Watanabe spigot (EWS; Novatech, Grasse, France) and *N*‐butyl‐2‐cyanoacrylate (NBCA; B. Braun, Melsungen, Germany).

## CASE REPORTS

### Case 1

**FIGURE 1:**
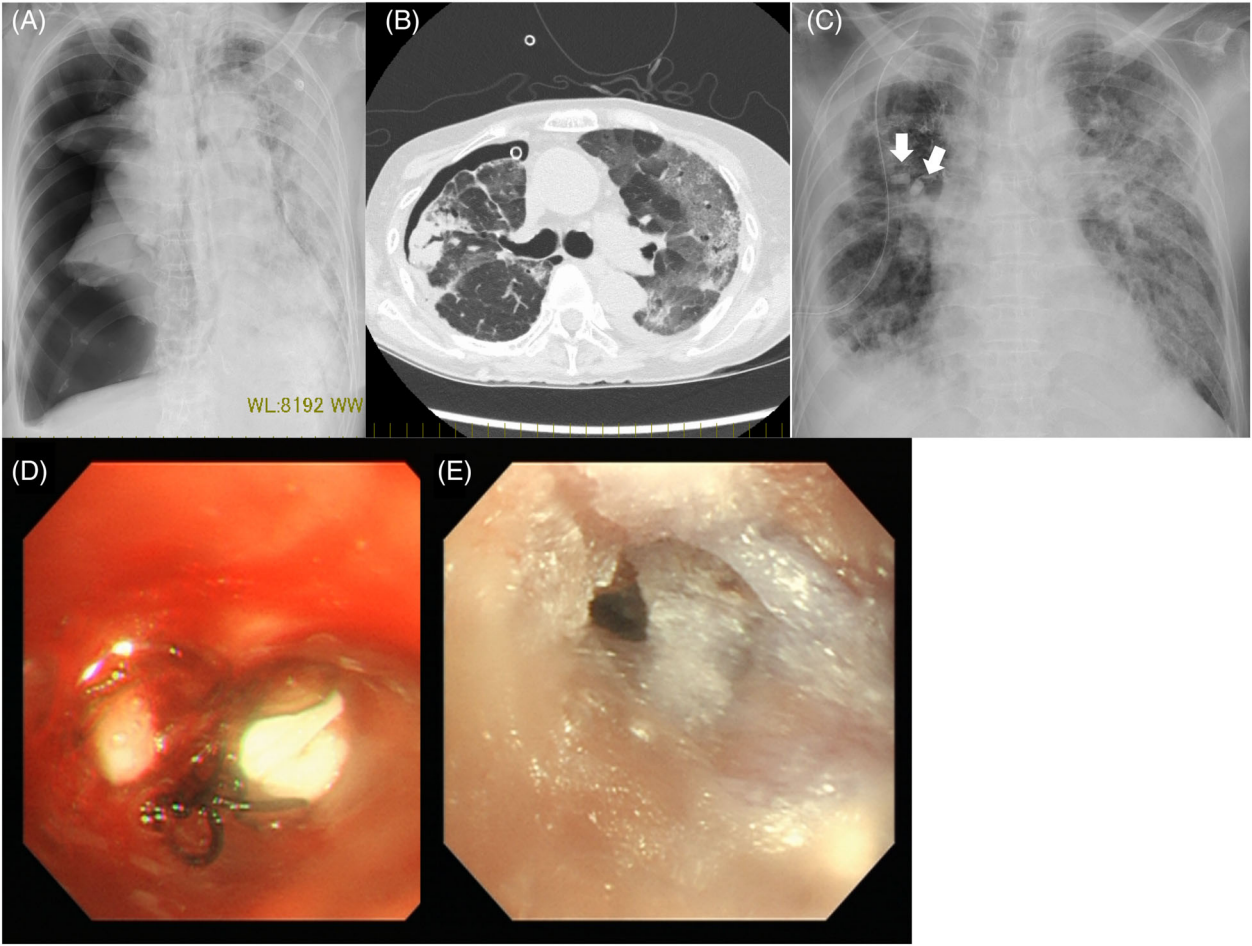
Case 1. (A) Large pneumothorax in the right lung. (B) Ground‐glass opacity was observed in both lungs. (C) An endobronchial Watanabe spigot (EWS) was inserted in the right lung (arrows). (D) EWS was inserted in the right B3a and B3b. The black thread attached to the EWS was used to remove the EWS. (E) *N*‐butyl‐2‐cyanoacrylate was applied to the entire B3

A 77‐year‐old woman with comorbid bronchiectasis was admitted to hospital with fever. The patient tested positive on a polymerase chain reaction test to SARS‐CoV‐2. On admission, the patient's body temperature was 37.3°C and her S_p_ o_2_ was 86% (nasal cannula, 2 L O_2_/min). Treatment was started with dexamethasone 6.6 mg and remdesivir, but the patient's respiratory condition worsened on Day 4 of admission, requiring intubation. The dose of methylprednisolone was increased to 120 mg/day and gradually decreased thereafter. The patient's respiratory condition improved, and she was extubated after 5 days of intubation. However, 4 days after extubation, the patient's respiratory condition rapidly deteriorated, with a chest x‐ray revealing right‐sided pneumothorax (Figure 1A). A 20‐Fr chest drain was inserted, and the lung was re‐expanded. However, air leakage persisted, despite repeated autologous blood pleurodesis.

Based on the ground‐glass shadows typical of *Pneumocystis* pneumonia (PCP) on chest computed tomography (CT; Figure 1B), elevated β‐d‐glucan and a history of long‐term steroid use, we considered PCP to be highly probable, although it was not proven. The patient was also positive for *Aspergillus* antigen, and we concluded that she had COVID‐19‐associated pulmonary aspergillosis (CAPA). The patient was treated with sulfamethoxazole/trimethoprim and antifungal drugs.

The patient was in poor general condition because of the complications associated with the infection. Surgery for pneumothorax was difficult, and thus we planned to insert an EWS. To identify the bronchus responsible for the pneumothorax, a balloon occlusion test was performed under bronchoscopy (balloon test), with decreased leakage found at B3. A 6‐mm EWS bronchial occlusion of B3 was performed, and 1 ml of a 1:1 mixture of lipiodol and NBCA was sprayed throughout B3 (Figure 1C–E). Because the leakage disappeared, the drains were removed on Day 3 after the procedure.

### Case 2

A 52‐year‐old man with a history of alcoholic liver disease was transferred to St. Luke's International Hospital because of bilateral pneumonia on chest x‐ray with an S_p_ o_2_ of 90%. The COVID‐19 antigen was detected, leading to a diagnosis of COVID‐19. The patient was started on dexamethasone 6.6 mg and remdesivir, but his respiratory condition worsened. The patient was then managed with a high‐flow nasal cannula (HFNC), with methylprednisolone 120 mg/day administered for 1 week starting on Day 7 of admission and then tapered. The patient's respiratory status gradually worsened, but he refused tracheal intubation; therefore, we continued HFNC therapy.

**FIGURE 2:**
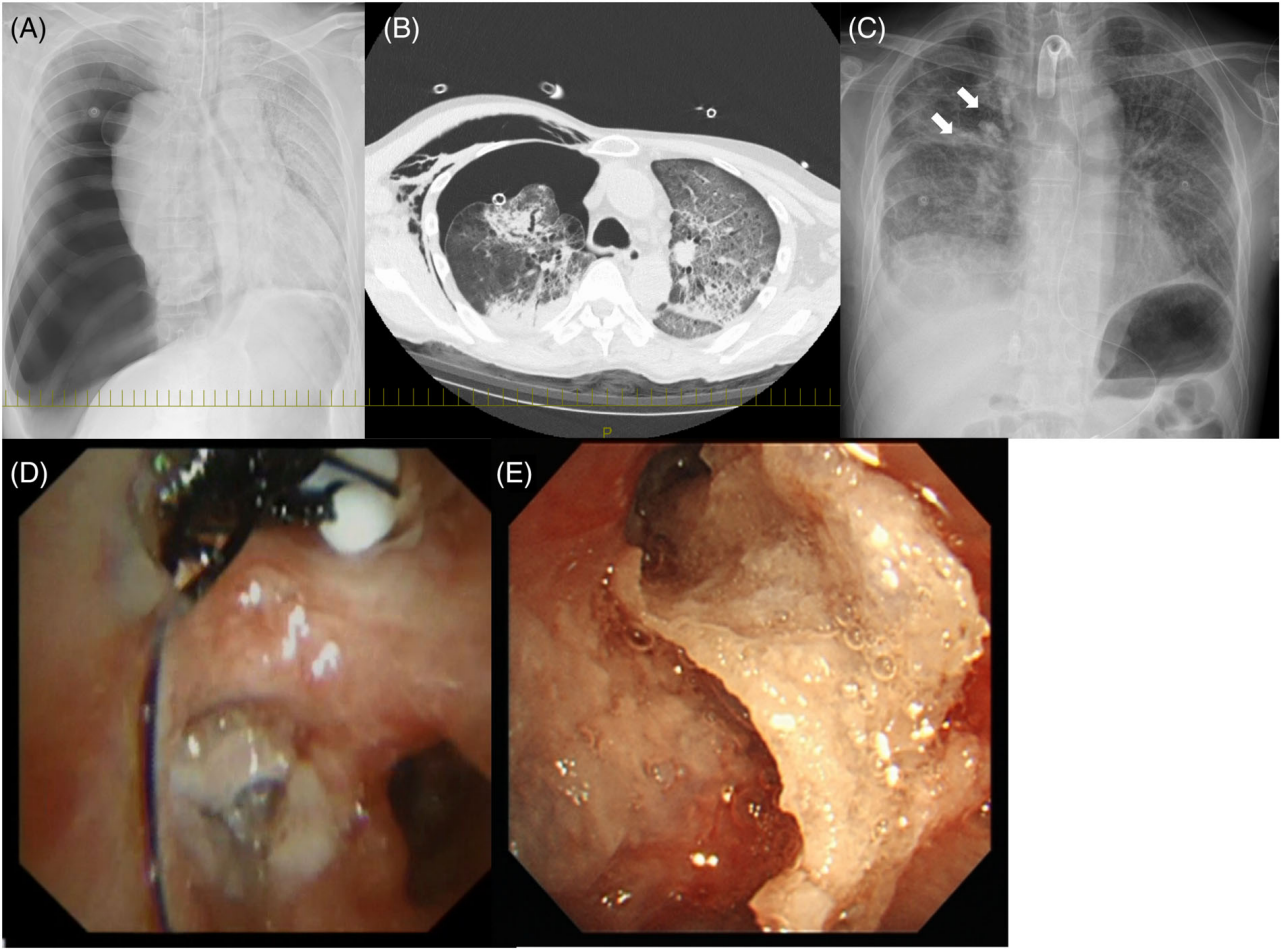
Case 2. (A, B) Large pneumothorax and subcutaneous emphysema in the right lung in a patient with coronavirus disease 2019 pneumonia. (C) An endobronchial Watanabe spigot (EWS) was inserted in the right lung (arrows). (D) Two EWSs were implanted in the right B1 and B3. (E) *N*‐butyl‐2‐cyanoacrylate was applied in B1 and B3

On Day 17 of hospitalization, the patient's respiratory condition suddenly deteriorated, and he was intubated. Chest x‐ray revealed right‐sided pneumothorax (Figure 2A,B) and a chest drain was inserted. The lungs were expanded, but air leakage persisted. Pleurodesis was attempted with autologous blood and 50% glucose, but there was no improvement. The patient's general condition was not conducive to surgery, and we attempted embolization with an EWS. A balloon test was performed using a scope with an outer diameter of 6 mm, but the scope could not be inserted into B1, and the amount of leakage did not decrease in B2. Because the leak volume was decreased by a balloon test in all of B3, 6‐ and 7‐mm EWSs were inserted into B3 and sprayed with 1 ml of a 1:1 mixture of lipiodol and NBCA for occlusion. After the procedure, the patient's air leakage worsened, and treatment with dextrose the next day did not lead to an improvement. A second attempt at embolization was performed using a scope with an outer diameter of 4 mm. B1 was occluded by bronchoscopy with an EWS and sprayed with 1.5 ml NBCA; B3 was sprayed with 0.5 ml NBCA. The level of air leakage decreased and 50% dextrose pleurodesis was performed the next day (Figure 2C–E). After the leak was completely stopped, the drain was removed 3 days after EWS implantation.

## DISCUSSION

Here, we report, for the first time, two cases of refractory pneumothorax caused by COVID‐19 pneumonia that were treated with EWS and NBCA.

Pneumothorax is reported to be an independent risk factor for death in COVID‐19.^3^ Multiple mechanisms are involved in the development of pneumothorax. A previous study reported that COVID‐19 causes cystic changes on CT,^4^ and tissue fragility may be the underlying cause. In the two cases reported in this study, cystic changes that were not present on admission appeared on subsequent CT. It is also known that patient self‐inflicted lung injury can be caused by increased tidal volume during spontaneous breathing and increased negative pressure in the thoracic cavity.^5^ A report from Italy documented an increase in pneumothorax during the second compared with first COVID‐19 wave, suggesting that the use of dexamethasone was responsible.^6^ These factors may contribute to the development of pneumothorax in both ventilated and non‐ventilated patients.

In patients with inoperable pneumothorax, treatment options for persistent air leaks include sealants, EWS, metal coils and endobronchial valves.^7^ Using an EWS has been reported to stop air leaks and avoid surgery in 77.6% of patients in whom surgery is difficult.^8^ NBCA has also been recently reported to be useful in combination with coils in patients with postoperative bronchopleural fistula.^9^ We used an EWS in combination with NBCA for more reliable occlusion. We were unable to find any previously published reports of the use of EWS and NBCA in combination for the treatment of persistent air leaks. At St. Luke's International Hospital, we use NBCA in combination with EWS after ethics committee review and patient consent.

Two previous publications have described the lack of efficacy of chest drainage in the treatment of pneumothorax attributable to COVID‐19 pneumonia. In the case of an 80‐year‐old man with a history of diabetes, surgery was considered high risk, so pleurodesis with autologous blood was performed, which resulted in improvement.^10^ In another series, thoracoscopy and bleb resection were performed in a 56‐year‐old man who was a smoker and a 70‐year‐old man with no previous medical history; both cases improved.^11^

In the present two cases, there was no improvement even after the insertion of a chest drain or pleurodesis. The first patient was elderly and in poor general condition, including the suspected coexistence of PCP and CAPA, and the second patient had a history of alcoholism and respiratory failure because of COVID‐19. After discussion with a thoracic surgeon, it was decided that surgery in these patients using general anaesthesia and one‐lung ventilation carried a high risk.

In pneumothorax caused by COVID‐19, more than one bronchus may be responsible for air leakage because COVID‐19 causes extensive damage to the lungs. To ensure embolization of the responsible bronchus in the present two cases, NBCA was used in combination with EWS to fill the gap and prevent dropout.

There are several precautions to consider with this treatment. First, the infectivity of COVID‐19 is a problem in the acute phase. Because the virus can be isolated from airway specimens for 10 days in patients with mild or moderate disease,^12^ and for 15 days in patients with severe disease,^13^ we performed bronchoscopy after this period. Next, bronchial occlusion may cause obstructive pneumonia. Neither of the present cases was complicated by severe pneumonia. We are considering removal of the EWS and NBCA if the patient is stable in the long term. NBCA can be removed under bronchoscopy using grasping forceps. However, if removal is difficult for some reason, the EWS may be left in place.^8^

COVID‐19 remains a major problem globally, and the incidence of pneumothorax caused by COVID‐19 is expected to increase. Bronchial occlusion with NBCA and an EWS is effective for refractory pneumothorax caused by COVID‐19, and may be a good option for patients who are not suitable for surgery because of their advanced age or poor general condition.

## CONFLICT OF INTEREST

None declared.

## AUTHOR CONTRIBUTION

Chie Morita wrote and edited the manuscript. Naoki Nishimura reviewed the manuscript. All authors performed the EWS procedure and/or participated in patient care.

## ETHICS STATEMENT

The authors declare that appropriate written informed consent was obtained from the patients for publication of this case report and any accompanying images.

## Data Availability

The data that support the findings of this study are available from the corresponding author upon reasonable request.
